# Bilan d’hospitalisation du service de dermatologie-vénérologie du CHU Ibn Sina Rabat Maroc

**Published:** 2010-11-29

**Authors:** Fatima Ezzahra Lamchahab, Kawtar Beqqal, Bouchra Guerrouj, Ibtissam Khoudri, Karima Senouci, Badredine Hassam, Mohamed Ait Ourhroui

**Affiliations:** 1 Service de dermatologie-vénérologie, CHU Ibn Sina Rabat, Maroc

**Keywords:** Dermatologie, Epidémiologie, Hospitalisation, Santé Publique, Maroc

## Abstract

**Introduction:**

Le service de Dermatologie de l’Hôpital Ibn Sina a pour mission de donner les soins en matière de dermatologie et concourt au service public de la santé au Maroc. Le but de ce travail est d’établir le profil épidémiologique et diagnostique des dermatoses en milieu hospitalier à travers cinq ans d’activité d’hospitalisation.

**Méthodes:**

Etude rétrospective menée sur une période de cinq ans de janvier 2003 à janvier 2008 concernant tous les patients hospitalisés au service de dermatologie.

**Résultats:**

2033 patients avaient été hospitalisés pendant la période étudiée. L’âge moyen était de 40 ans. Les patients provenaient surtout de la région Rabat – Salé – Zaër. Chez l’adulte, les pathologies les plus fréquemment rencontrées étaient les maladies de système et les pathologies infectieuses. Le taux de mortalité était très faible. Les dermatoses du milieu hospitalier sont diverses. Les maladies de système occupent le premier rang. Ceci peut être justifié par la multitude des bilans nécessaires. Les dermatoses bulleuses nécessitent une longue prise en charge et sont à l’origine des séjours prolongés des patients.

**Conclusion:**

Les affections cutanées constituent une source majeure de morbidité. Les formes graves nécessitent une prise en charge adéquate.

## Introduction

Le service de Dermatologie de l’Hôpital Ibn Sina a été créé en 1969. Il a pour mission de donner les soins en matière de dermatologie et concourt au service public de la santé au Maroc. Cette mission se résume à des consultations, des soins et des hospitalisations des malades présentant une pathologie dermatologique. Peu d’études se sont intéressées à l’activité hospitalière en dermatologie [1-6].

Le but de ce travail est d’établir le profil épidémiologique et diagnostique des dermatoses en milieu hospitalier au service de dermatologie du centre hospitalier universitaire Ibn Sina Rabat.

## Méthodes

Il s’agit d’une étude mono-centrique rétrospective menée sur une période de 5 ans de janvier 2003 à janvier 2008 concernant tous les patients hospitalisés au service de dermatologie vénérologie du Centre Hospitalier Universitaire Ibn Sina Rabat au Maroc. Ce service a une capacité litière estimée à 31 lits. L’activité d’hospitalisation a été décrite à partir des dossiers archivés et du registre des patients hospitalisés. Nous avons précisé pour chaque patient; l’âge, le sexe, l’origine géographique, le motif d’hospitalisation, l’évolution, la durée et la période d’hospitalisation.

## Résultats

**Taille de l’échantillon**

**Figure 1:**
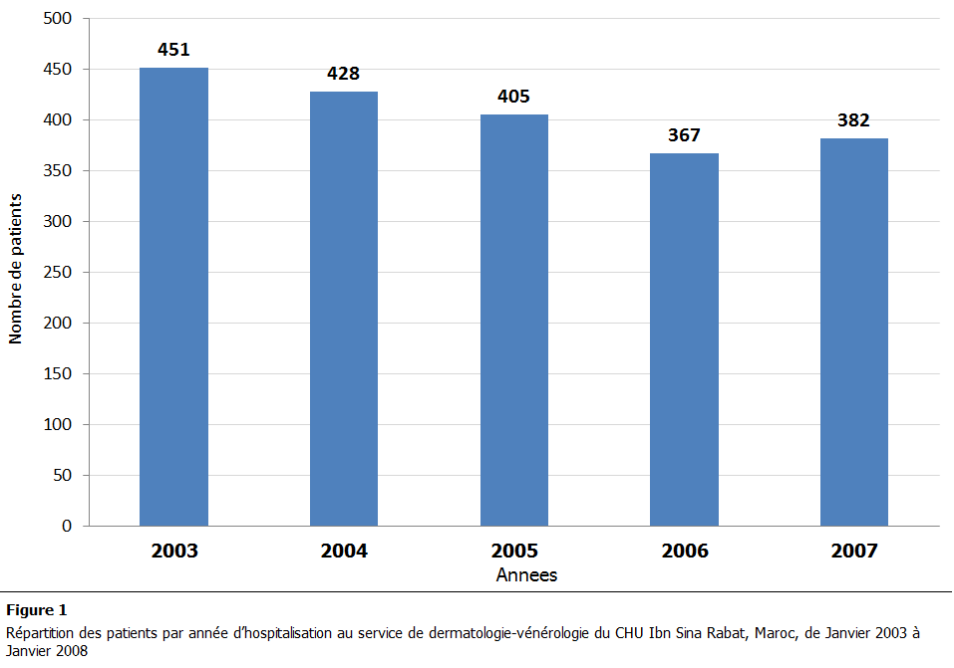
Répartition des patients par année d’hospitalisation au service de dermatologie-vénérologie du CHU Ibn Sina Rabat, Maroc, de Janvier 2003 à Janvier 2008

2033 patients avaient été hospitalisés pendant la période étendue de janvier 2003 à janvier 2007; 451 patients en 2003, 428 patients en 2004, 405 patients en 2005, 367 patients en 2006 et 382 patients en 2007 (Figure 1).

**Caractéristiques démographiques**

**Figure 2:**
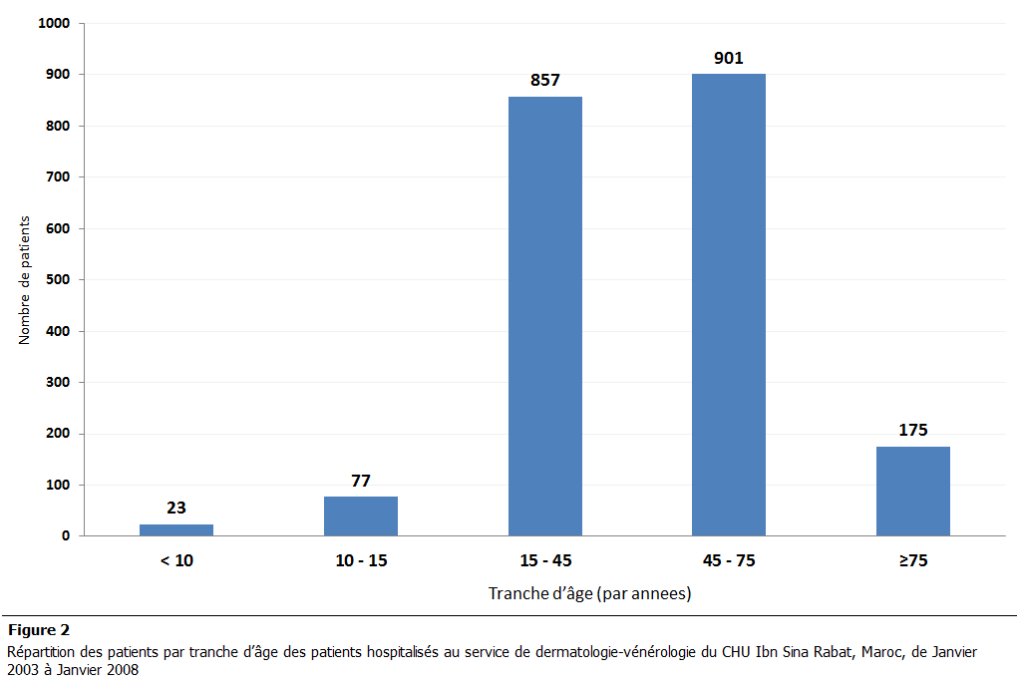
Répartition des patients par tranche d’âge des patients hospitalisés au service de dermatologie-vénérologie du CHU Ibn Sina Rabat, Maroc, de Janvier 2003 à Janvier 2008

Une légère prédominance féminine avait été notée avec 1011 hommes (49.7%) et de 1022 femmes (50.3%). Le sex ratio était de 0.98. L’âge moyen des patients était de 40.05 ans avec des extrêmes de 1 an à 95 ans. Les patients âgés de moins de 15 ans représentaient 4.91% des patients hospitalisés, soit 100 enfants hospitalisés pendant la période étudiée. La tranche d’âge la plus concernée était de 45 à 75ans avec un taux de 44.31% suivie de la tranche de 15 à 45 ans avec un taux de 42.13%. Les patients âgés de plus de 75 ans ne représentaient que 8.65% (Figure 2). Le taux de mortalité était de 0.025% (n: 10).

**Caractéristiques des hospitalisations**

La durée d’hospitalisation moyenne était de 20 jours pour l’ensemble des malades et variait de 2 à 42 jours; elle était plus longue pour les dermatoses bulleuses (65 jours). Le nombre moyen d’hospitalisation était de 1.13 patients par jour. La médiane des patients hospitalisés variait en fonction de la saison, on a choisi trois périodes saisonnières, la première comprenait les mois janvier, février, mars et avril. La deuxième comprenait les mois mai, juin, juillet et août. La troisième comprenait les mois septembre, octobre, novembre et décembre. Ainsi, la première période était caractérisée par un nombre accru d’hospitalisations estimé à 719 sur les cinq ans (35.63%), plus marqué au mois d’Avril avec 203 hospitalisations (10%). Le nombre d’hospitalisations le plus bas était observé à la troisième période et surtout au mois d’Août avec seulement 89 hospitalisations pendant la période étudiée soit 4.37%.

Les patients provenaient principalement de la région Rabat – Salé – Zaër avec un taux 37.65% (n: 763), mais également d’autres régions du royaume notamment la région Gharb-Chrarda-Beni Hssen avec 17.17%, la région Grand Casablanca avec 7.73%, la région Meknès-Tafilalet avec 5.23% et la région Tanger-Tétouan avec 4.77%. Le taux de mortalité estimé à 0.49% des hospitalisations du service de dermatologie soit 10 patients décédés pendant les 5 années étudiés.

**Les motifs d’hospitalisation**

Les pathologies retrouvées étaient réparties en différents groupes comme suit (tableau 1).

Les maladies de système représentaient 19.42% des motifs d’hospitalisations (n: 395). Le lupus dans toutes ses formes, occupait le premier rang avec un taux de 33.92% des maladies de système (n: 134), suivie de la maladie de Behçet (n: 97), puis la sclérodermie (n: 74), la sarcoïdose (n: 63) et d’autres maladies de système y compris les dermatomyosites, le syndrome de Gougerôt Jögren, les connectivites mixtes.

La pathologie infectieuse occupait le deuxième rang (n: 306), soit 15.05% des hospitalisations, infections bactériennes à type d’érysipèle (n: 99) soit 32.35% des pathologies infectieuses, tuberculose cutanée (n: 64), lèpre (n: 4), infections parasitaires (n: 19) dont 14 cas de leishmaniose, infections virales (n: 13) avec 8 nouveaux cas de SIDA, infections fongiques (n: 10) y compris 8 cas de mycoses profondes.

Les états d’hypersensibilité constituaient 12.79% des hospitalisations (n: 260) et comprenaient la dermatite atopique, les eczémas, le prurit chronique, l’urticaire, le prurigo, le lichen, le syndrome de sweet et d’autres.

Les dermatoses bulleuses représentaient 10.82% des hospitalisations dominés par le pemphigus (n: 140) soit 63.63% des dermatoses bulleuses, la pemphigoide gestationis (n: 38), la pemphigoide bulleuse (n: 31) et d’autres dermatoses bulleuses plus rares.

La pathologie tumorale occupait la cinquième place, (n: 134) soit 6.59% des hospitalisations, lymphomes cutanées (n: 46) soit 34.33% des pathologies tumorales, carcinomes spinocellulaires (n: 27), carcinomes basocellulaires (n: 22), mélanomes (n: 18) et d’autres tumeurs malignes plus rares ou bénignes.

La pathologie vasculaire représentait 5.36% des motifs d’hospitalisation (n: 109) dominée par la maladie de kaposi (n: 47) soit 43.11% des pathologies vasculaires, l’ulcère de jambe (n: 40), et d’autres (purpura, livédo, vascularite).

Les dermatoses inflammatoires représentaient 5.16% des hospitalisations (n: 105), principalement le psoriasis dans ses formes graves (n: 91).

Les génodermatoses constituaient 4.52% des hospitalisations (n: 92), principalement le xéroderma pigmentosum (n: 54) soit 54.34% des génodermatoses, les neurofibromatoses (n: 17) et d’autres plus rares (25 cas).

Les toxidermies représentaient 2.09% des hospitalisations avec 59 cas.

D’autres dermatoses sont plus rares; les dermatoses psychosomatiques principalement la pathomimie: (n: 10), les maladies des annexes, la pelade (n: 17), les dermatoses faciales; la rosacée (n: 14) et l’acné (n: 2), les troubles de la pigmentation (n: 4) incluant le vitiligo (n: 2). Et d’autres étiologies diverses (n: 221) (chéilite, photodermatoses, brûlures cutanées, escarres, maux perforants plantaires…).

## Discussion

Les affections cutanées constituent un problème majeur de santé publique dans les pays en voie de développement, notamment au Maghreb. En effet au Maroc est, sous les effets conjugués de l’environnement, des conditions de vie et des influences culturelles, au carrefour de toutes les pathologies.

L’histoire et la renommée de l’hôpital Ibn Sina font de cet établissement, à côté des autres CHU du Royaume, un centre de référence de soins et de consultations de rayonnement national. Ainsi, notre service continue de recevoir des patients de toutes les régions du Maroc. Notre étude est originale et montre que l’activité hospitalière du service de dermatologie est importante, tant quantitativement que par l’importance des pathologies prises en charge, en santé publique.

Les dermatoses du milieu hospitalier sont diverses et variées et constituent une source majeure de morbidité. Tous les types de pathologies dermatologiques sont pris en charge dans notre formation. Quoique, les pathologies cutanées notées restent certes sous-estimées, vu que celles-ci ne motivent des consultations dans notre contexte que si elles occasionnent une gêne fonctionnelle ou un préjudice esthétique. Une étude [1] menée dans un service de dermatologie au Togo a conclu que les principales affections justifiant une hospitalisation étaient par ordre décroissant de fréquence les toxidermies graves, la maladie de Kaposi, les connectivites, le zona et de l’érysipèle.

D’autres études occidentales [2-6] ont rapporté cette diversité des dermatoses au milieu hospitalier, représentaient principalement par les néoplasies, le psoriasis et les ulcères chroniques. Ce profil varié des dermatoses notées dans ces différentes études est du aux variations ethniques, géographiques, culturelles et socio-économiques entre le Maroc, les pays occidentaux et subsahariens. De plus, les différences de méthodes de mesures et d’échantillonnage font que les comparaisons entre les études soient difficiles.

Le temps de séjour dépend de la gravité des pathologies hospitalisées. Il est allongé chez les patients ayant présenté des complications, infectieuses et thromboemboliques, notamment chez les patients sous forte dose de corticothérapie.

Notre étude montre une légère prédominance féminine avec un sex-ratio de 0.98. Il s’agissait d’adultes jeunes avec un âge moyen de 40 ans. Un nombre faible de dermatoses pédiatriques fut observé, ceci est dû au mode de recrutement. Puisque l’hôpital d’enfants recrute le maximum des enfants malades. Le taux de mortalité était très faible (0.025%) en comparaison avec le taux de mortalité globale des autres services médicaux du CHU, étant de 0.55% sur 39141 patients hospitalisés dans les différents services médicaux, soit 218 patients décédés. Ainsi, les différentes dermatoses observées engagent rarement le pronostic vital des patients.

Concernant les principaux motifs d’hospitalisation, les maladies de système occupaient le premier rang dans notre contexte, ceci peut être justifié par la difficulté diagnostique, la chronicité des pathologies et par la multitude des bilans nécessaires. Les maladies de système sont dominées par le lupus qui est une maladie systémique polymorphe, les manifestations cutanées sont fréquentes et pouvant être rebelles et révélatrices de la maladie [7,8]. De nombreux facteurs génétiques, endocriniens, immunologiques et d’environnement contribuent au déclenchement puis à l’entretien de la maladie [8].

La pathologie infectieuse était également fréquente. Les infections bactériennes occupent une place importante. Ces infections sont favorisées par une hygiène défectueuse, des conditions socioéconomiques médiocres et par l’insuffisance des soins médicaux [9]. La contagiosité des dermatoses bactériennes conduit souvent à leur extension par auto inoculation manu-portée (visage et zones découvertes) ou par macération locale (sous les couches chez le nourrisson) et parfois à de petites épidémies en particulier chez l’enfant en collectivité (crèches, écoles) par hétéro-inoculation.

L’érysipèle constituait un motif courant d’hospitalisation, est favorisé par l’effraction de l’intégrité de la peau, la mauvaise hygiène et l’immunodépression [9,10].

La tuberculose sévit selon le mode endémique au Maroc et constitue un problème de santé public dans notre pays. Cette fréquence pourrait être expliquée par les conditions climatiques de certaines régions du royaume, les conditions d’hygiènes défavorables, la contagiosité et la dénutrition [11,12]. Pour cela le ministère de la santé a lancé un plan national 2006-2015 de lutte contre la tuberculose au Maroc.

Malgré le caractère bénin de la plupart de ces infections, leur prise en charge efficace et immédiate est indispensable pour éviter certains incidents et complications qui peuvent être graves voire mortelles : septicémies, état de choc septique, récidive ou extension des lésions infectieuses, propagation des infections contagieuses dans une collectivité.

La leishmaniose, fréquente dans notre pays. Bien que bénigne elle nécessite un traitement intra hospitalier par l’antimoniate de méglumine [13]. En effet, les leishmanioses sont diverses et complexes pour plusieurs raisons : la multiplicité et la complexité des cycles parasitaires, la diversité des réservoirs du parasite et la distribution géographique différente; la leishmaniose cutanée à leishmania major sévit dans les zones présahariennes désertiques sur un mode endémo-épidemique, à *Leishmania tropica* dans les régions semi-aride au centre du pays dans des foyers hypoendémiques et à *Leishmania infuntum* dans le nord du pays sous un mode hypoendémique dans les foyers ruraux dispersés [13-15]. Les formes cliniques sont nombreuses et variées et peuvent se présenter sous forme: d’ulcérations torpides indolores, parfois surinfectées, des formes papulo-nodulaires ou nodulaires pures, papulo-squameuses et ulcéro-végétantes [15]. La forme la plus rencontrée reste cependant la forme ulcérée. Le programme de lutte contre la leishmaniose établie par le Ministère de la Santé publique a permis une nette diminution des cas [14].

Quatre cas de lèpre avaient été colligés. La stratégie de lutte contre la lèpre est essentiellement une stratégie de prévention secondaire. Elle est basée sur la détection et le traitement des malades afin de baisser la charge hansénienne et rompre la chaîne de transmission. Devant les progrès réalisés avec la polychimiothérapie (PCT), l’OMS a lancé en 1991 la stratégie d’élimination de la lèpre en tant que problème de santé publique [16]. Dans ce cadre, le protocole thérapeutique fondé sur les polychimiothérapies a été conçu pour remédier à l’apparition de plus en plus fréquente d’une résistance primaire et secondaire à la monothérapie (Dapsone).

La fréquence des dermatoses mycosiques (complexe des plis) chez nos patients peut s'expliquer par nos conditions climatiques et aussi par les pratiques religieuses (les ablutions). Ainsi, il est primordial d’améliorer les conditions d’hygiène des patients et multiplier les efforts épidémiologiques (compagnes de vaccinations antituberculeuse, prévention VIH, programme d’éradication de la lèpre…). Il est aussi indispensable de lutter contre les microorganismes nosocomiaux souvent multi résistants et difficiles à traiter.

Les états d’hypersensibilité comprenaient la dermatite atopique, les eczémas, le prurit chronique, l’urticaire, le prurigo, le lichen, le syndrome de sweet et d’autres. Le prurit chronique, motif fréquent de consultation, occupait une place importante dans notre étude. Les principales causes du prurit paranéoplasique sont les hémopathies malignes, le cancer du sein chez la femme et les cancers pulmonaires et prostatiques chez l’homme [17].

Les dermatoses bulleuses étaient dominées par le pemphigus, fréquent dans notre pays et nécessitant un séjour prolongé du fait de la difficulté thérapeutique et des complications infectieuses et iatrogènes [18,19].

La pathologie tumorale était dominée dans notre contexte par les lymphomes cutanés et les mélanomes nécessitant un bilan d’extension, ce qui augmente la durée de séjour des patients. Concernant les carcinomes basocellulaires et les carcinomes spinocellulaires sont fréquents mais essentiellement vues en ambulatoire. Des compagnes d’information et de dépistage des sujets à risque s’avèrent ainsi nécessaires. D’où l’intérêt des journées de sensibilisation, organisées annuellement par notre service, afin de sensibiliser la population générale sur les méfaits du soleil et les moyens de photoprotection [20].

Les toxidermies représentaient 2.09% des hospitalisations. Il s’agit le plus souvent des cas de DRESS, rash maculopapuleux voire plus graves à type de syndrome de Stevens Johnson et de Lyell transférés de la réanimation après stabilisation de leur état et pour complément de prise en charge. Elles posent le problème de l'imputabilité chez des sujets le plus souvent polymédicamentés. Les médicaments les plus incriminées sont les antibiotiques, les anticonvulsivants, les inhibiteurs de l’enzyme de conversion et l’allopurinol [21].

Parmi les cas de génodermatoses hospitalisés, le xéroderma pigmentosum en constitue un motif fréquent. Cette maladie nécessite une prise en charge multidisciplinaire étroite, incluant l’éducation des jeunes patients et de leur entourage ainsi que le traitement des tumeurs cutanées [22]. D’autres dermatoses bien que fréquentes tel que le psoriasis, l’acné et le lichen avaient été rarement rapportées dans cette étude. En raison de leur caractère bénin, ces affections nécessitent rarement l’hospitalisation, sauf dans les formes graves.

## Conclusion

L’activité hospitalière constitue une activité importante au service de dermatologie. Les affections cutanées constituent une source majeure de morbidité et présentent un impact important sur la qualité de vie des patients, vu le préjugé esthétique. Les maladies de système, les dermatoses infectieuses et les dermatoses bulleuses constituent les principaux motifs d’hospitalisation de notre service. D’où l’intérêt d’établir des stratégies de dépistage précoce, à l’échelon national, afin d’assurer un prise en charge adéquate.

## Conflits d’intérêts

Les auteurs ne déclarent aucun conflit d’intérêts

## Contribution des auteurs

Lamchahab Fatima Ezzahra: Rédaction scientifique, exploitation des dossiers et analyse statistique des données, Beqqal Kawtar: Participation à la saisie des données, Ibtissam Khoudri et Bouchra Guerrouj: Membres de la rédaction scientifique, Karima Senouci, Hassam Badredine: Supervision du travail, Mohamed Ait Ourhroui: Direction de la rédaction scientifique.

## Figures and Tables

**Tableau 1:** Distribution des motifs d’hospitalisation au service de dermatologie -vénérologie du CHU Ibn Sina Rabat, Maroc, de Janvier 2003 à Janvier 2008

**Motif d’hospitalisation**	**Fréquence (%)**
Maladies de système	19,42
Pathologies infectieuses	15,05
Etats d'hypersensibilité	12,79
Dermatoses bulleuses	10,82
Génodermatoses	4,52
Pathologies vasculaires	5,36
Pathologies tumorales	6,59
Toxidermies	2,09
Dermatoses inflammatoires	5,16
Dermatoses psychosomatiques	0,49
Dermatoses faciales	0,78
Troubles de la pigmentation	0,20
Maladies des annexes	0,09
Autres	10,8
